# Bilateral breast implant associated chronic lymphocytic leukemia/small lymphocytic lymphoma (CLL/SLL): A case report

**DOI:** 10.1016/j.ijscr.2020.05.039

**Published:** 2020-05-29

**Authors:** Patrick P. Bletsis, Laura E. Janssen, Otto Visser, Saskia R. Offerman, Michiel A. Tellier, Laurens Laterveer, Peter Houpt

**Affiliations:** a Faculty of Medical Sciences, University of Groningen, Groningen, the Netherlands; b Department of Plastic and Reconstructive Surgery, Isala Clinics, Zwolle, the Netherlands; c Department of Hematology, Isala Clinics, Zwolle, the Netherlands; d Department of Pathology, Isala Clinics, Zwolle, the Netherlands

**Keywords:** Case report, Small lymphocytic lymphoma, Chronic lymphocytic leukemia, B-cell lymphoma, Breast implant

## Abstract

**Introduction:**

An estimated 30.000 breast implants are placed in the Netherlands annually. An increasing amount of reports have linked implants to the rare anaplastic large cell lymphoma (ALCL). Other implant-related lymphomas, such as those of B-cell lineage, are much rarer.

**Presentation of case:**

A 62-year-old female presented with pain and Baker grade III capsular contraction of the right breast. Subpectorally placed textured anatomical implants had been in situ for 26 years after cosmetic augmentation. Magnetic Resonance Imaging (MRI) showed bilateral implant leakage. Explantation of both implants confirmed bilateral leakage after which symptoms went into remission. Three months later our patient noticed an erythematous area, scar swelling and serous fluid leakage on the lateral side of the inframammary fold of the right breast. Siliconomas were excised bilaterally together with a partial capsulectomy on the left. Histopathology and immunohistochemical analysis showed monotonous small cell B-lymphocytic infiltration (CD20+, CD5+, CD23+, ALK-) in both capsules, highly suggestive for chronic lymphocytic leukemia (CLL)/small lymphocytic lymphoma (SLL).

**Discussion:**

CLL/SLL are classified as nearly the same disease. The primary difference is the localization; CLL is found the bone marrow and blood whereas SLL is predominantly in the lymph nodes and spleen. There are no previous descriptions of bilateral CLL/SLL found in periprosthetic capsules.

**Conclusion:**

Breast implants are increasingly linked to various malignancies. In most cases, including our patient, implant explantation together with long-term follow-up suffices. MRI yields additional value in early stage diagnosis. More research is required to further optimize multidisciplinary care and improve patient outcomes.

## Highlights

• Implant-associated breast lymphomas are rare and most are of T-cell lineage.
• CLL is found the bone marrow and blood whereas SLL is predominantly found in the lymph nodes and spleen.
• Explantation of the implant induces remission of most breast implant-associated lymphomas.
• Follow-up complete blood count and immunophenotyping is advised every three months (or earlier in case of new symptoms).

## 1 Introduction

Over the years, breast implants have acquired a prominent role in the enhancement of breast aesthetics for reconstructive and cosmetic purposes. It is estimated that between 25.000 and 30.000 breast implants are placed in the Netherlands annually [1]. Since the 1990’s, an increasing amount of reports have linked a rare type of T-cell lymphoma to breast implants. This breast implant associated anaplastic large cell lymphoma (BIA-ALCL) is extremely rare, however, it has been suggested that some relationship exists [2]. Other lymphomas, such as B-cell associated are even rarer. The fourth edition of the World Health Organization Classification of Tumours of Haematopoietic and Lymphoid Tissues classifies chronic lymphocytic leukemia (CLL) as almost the same disease as small lymphocytic lymphoma (SLL) [3]. Both are non-Hodgkin’s lymphoma subtypes originating from the B-cell lineage. The primary difference is the localization of leukemic cells; in CLL the majority is found in the bone marrow and blood as opposed to SLL where they are predominantly found in the lymph nodes and spleen. Previous literature has described CLL/SLL as primary lymphoma of the breast but usually in combination with other malignancies such as of the breast [4]. To our best of knowledge there are no previous descriptions of CLL/SLL found bilaterally in periprosthetic capsules. This article was composed in accordance with the SCARE guidelines [5]. This study was exempted of ethical approval by the hospital’s medical ethics committee.

## 2 Case report

**Fig. 1:**
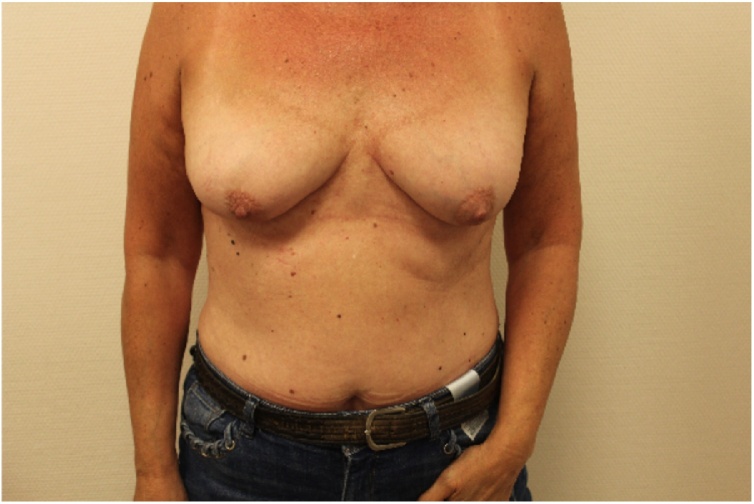
Frontal view after explantation of the breast implants in our 62-year-old patient.

**Fig. 2:**
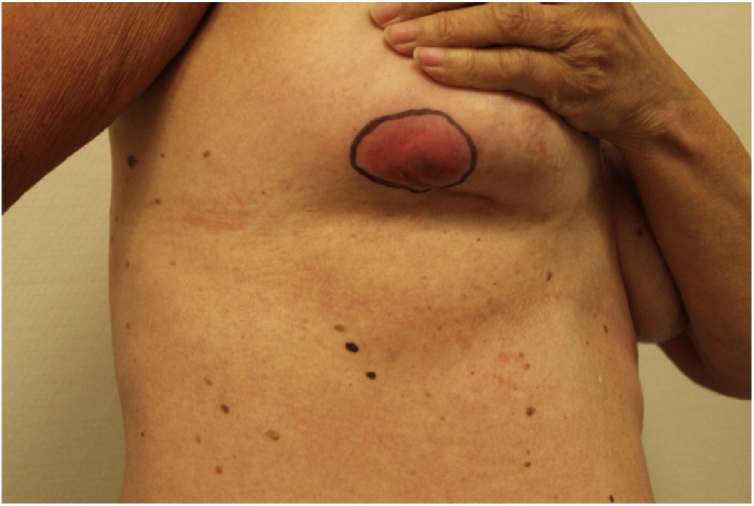
Swollen erythematous area on the lateral side of the inframammary fold of the right breast.

**Fig. 3:**
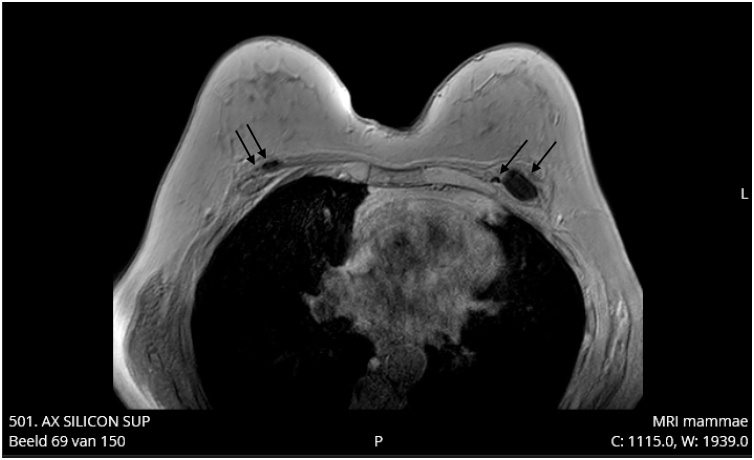
Silicone residuals after explantation of the breast implants on protocol breast MRI. The diameter of the largest silicone pocket (left) is 2.3 × 1.5 cm.

**Fig. 4:**
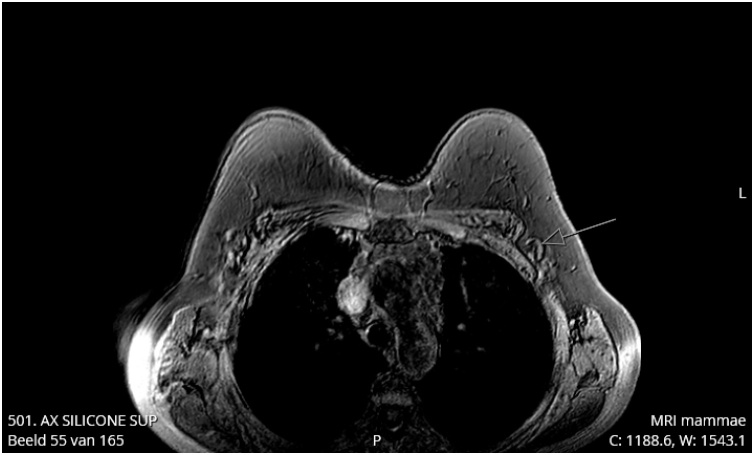
Follow-up breast MRI after three months. No evident identifiable pathologies such as macro level siliconomas as seen on earlier MRI. Possibly one lateral lymph node (left) with minimal silicone residuals.

**Fig. 5:**
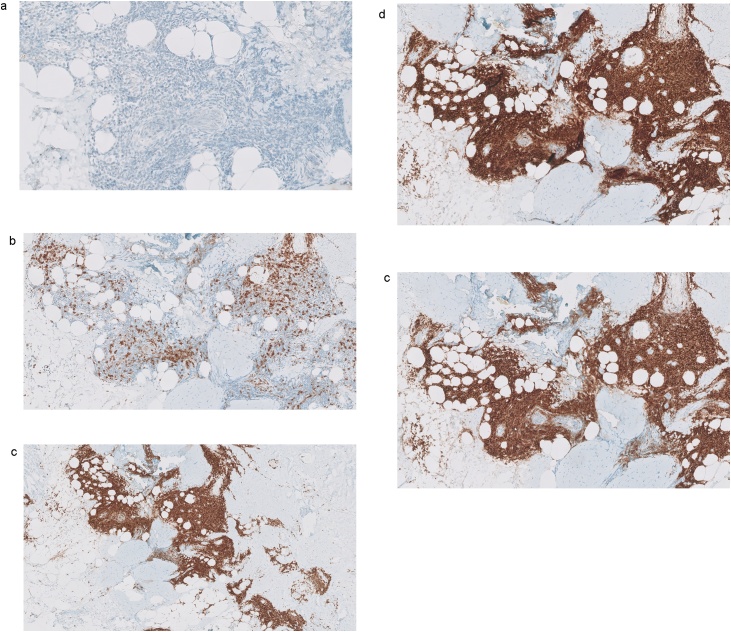
a) Immunohistochemistry (ALK-staining) of tissue from the lateral and medial lower quadrant of the left breast of our 62-year-old patient. b) Immunohistochemistry (CD3-staining) of tissue from the lateral and medial lower quadrant of the left breast of our 62-year-old patient. c) Immunohistochemistry (CD5-staining) of tissue from the lateral and medial lower quadrant of the left breast of our 62-year-old patient. d) Immunohistochemistry (CD20-staining) of tissue from the lateral and medial lower quadrant of the left breast of our 62-year-old patient. e) Immunohistochemistry (CD23-staining) of tissue from the lateral and medial lower quadrant of the left breast of our 62-year-old patient.

In June 2018, a 62-year-old healthy Caucasian woman underwent a standard follow-up magnetic resonance imaging (MRI) of subpectorally placed textured anatomical breast implants [Allergan (Dublin, Ireland)] in situ for 26 years after cosmetic augmentation. She had never experienced any complaints related to the breast implants. In 1990, she was treated for a basal cell carcinoma on her left shoulder but has no other comorbidities except for past tobacco use. Her eldest son was diagnosed with non-Hodgkin’s lymphoma at age 12. In August 2018, the patient started experiencing pain and Baker grade III capsular contraction of the right breast. Control MRI showed breast implant leakage. Explantation of both implants in January 2019 showed bilateral leakage after which symptoms went into remission. About three months later our patient noted an erythematous area on the lateral side of the inframammary fold of the right breast (Fig. 1, Fig. 2). She recalled that the scar of this breast had been swelling increasingly followed by leakage of some serous fluid out of the scar. Although not ill she was prescribed oral antibiotics (amoxicillin clavulanate) for one week, without alleviating symptoms. Ultrasound echography of the right breast showed “snowstorm sign”, suggestive for residual silicone after leakage. Subsequently, the siliconomas and old scars were removed under local anesthesia and send for pathological assessment. The pathology report stated that the skin contained some macroscopic cystic abnormalities filled with a mucous substance. Microscopically extensive fibrosis was observed with round nucleate inflammatory cell infiltration, multinucleated giant cell macrophages and foreign material, likely silicone. Yet, the discomfort and inflammatory response continued to exist for which another MRI was made. This showed bilateral subpectoral residual silicone particles, with the largest silicone pocket of about 2,3 × 1,5 cm in the left breast (Fig. 3, Fig. 4). In June 2019, revision surgery was performed in order to remove the siliconomas. Several superficially located siliconomas were excised on the right side on the right side. On the left, a thickened capsule was found containing a substantial amount of silicone. Therefore, a partial capsulectomy was performed simultaneously removing the substance. Histopathology and immunohistochemical analysis showed monotonous small cell B-lymphocytic infiltration (CD20+, CD5+, CD23+, ALK-) in both capsules, highly suggestive for CLL/SLL (Fig. 5a–e). Breast implant associated anaplastic large cell lymphoma (BIA-ALCL) was considered but excluded as CD30 tested negative. Microbiology tested positive for *Staphylococcus epidermidis*. After diagnosis, the hematology department was consulted for systemic medical workup and further guidance. Complete blood count (CBC) indicated a mild hypogammaglobulinemia (0.32 g/L IgM and 5.6 g/L IgG) and leukocytosis (10.5 × 10^9^/L). In the peripheral blood there was an absolute number of 0.04 10e9/L (CD19+, CD20+, CD5+, Lambda+, CD23+, CD43+, CD45+, and CD200+) monoclonal B-cells. Abdominal ultrasound was made to investigate the spleen and abdominal lymph nodes while thoracic X-rays were made to assess the mediastinum and hilar lymph nodes; no significant abnormalities were reported. Subsequent bone marrow biopsy showed cell-rich tissue with large fields of atypical lymphocytes and grumulee pattern. Additionally, a low percentage of monoclonal B-cells (0.04 × 10e9/L) in the peripheral blood after explantation of the breast implants suggested either CLL/SLL localized in the bone marrow and periprosthetic capsule or induced by the prosthesis material. Time between follow-up appointments will gradually be extended to six months if the lymphoma remains in remission. No additional treatment was necessary. Follow-up CBC and immunophenotyping will take place every three months (or earlier in the case of new symptoms) and will be extended to six months if the lymphoma remains in remission. Bone marrow biopsy may be utilized in the future to follow-up on disease activity and the effect of explantation.

## 3 Discussion

Primary lymphomas of the breast are extremely rare and account for only 0.5 percent of all breast malignancies the majority of which are B-cell driven [6]. The association between B-cell lymphomas and implants was therefore previously discarded because of the low incidence (contrary to BIA-ALCL) and variability [7]. A literature search yielded previous documentations of breast implant associated B-cell lymphomas but none described CLL/SLL in an otherwise healthy person. Other types included: (1) follicular lymphoma [8], (2) primary effusion lymphoma [9], (3) lymphoplasmacytic lymphoma [10], (4) nodal marginal zone; follicular lymphoma [11], (5) large B-cell lymphoma [12], (6) intravascular large B-cell lymphoma [13], and (7) low-grade B-cell lymphoma [14]. Also, the majority were unilateral malignancies instead of bilateral as was the case with our patient. In 2015, a case report described CLL/SLL in a patient that had undergone alloplastic breast reconstruction after bilateral prophylactic mastectomy [15]. However, we believe it is noteworthy to mention that the patient suffered from Li-Fraumeni syndrome and germline mutations in TP53 and was also diagnosed with intraductal carcinoma and BIA-ALCL among other malignancies. Moreover, the authors did not mention whether the CLL/SLL manifested inside of the periprosthetic capsule and if so, its laterality.

Most lymphomas of the breast originate from B-cells and are frequently situated on the right side [16]. In our patient, monoclonal B-cell lymphoma was found bilaterally. Tsimberidou et al. investigated the occurrence of other malignancies with CLL/SLL [17]. The authors found that 324 (16 percent) out of 2.028 patients had a history of other malignancies while 227 (11.2 percent) developed one during the follow-up period. The most common ones being skin, prostate and breast malignancies (30,13 and 9 percent, respectively). Therefore, one may conclude that there could be a relationship between the past basal cell carcinoma and the more recently discovered CLL/SLL. Race has been shown to affect the incidence with people of Caucasian descent being at a higher risk [18]. The incidence of CLL/SLL in Asian countries such as China and Japan is estimated to be 10 percent of that in Western countries [19]. Familiar genetic predisposition has also been suggested to play a key role with first-degree family members being at a higher risk [20]. Goldin et al. found that 17 percent of first-degree family members of CLL patients also have monoclonal B-cell lymphocytosis [21]. Some studies have argued for genetic anticipation meaning that the disease develops at an earlier age in following generations [22,23]. This is consistent with the stories of our patient and her son who was diagnosed with non-Hodgkin’s lymphoma at age 12. To our knowledge no other family members have suffered from similar (haematological) malignancies or CLL/SLL specifically. Other factors such as (second hand) smoking and exposure to heavy solvents have been debated in the literature but have not (yet) been proven to increase the risk of CLL/SLL [24,25].

The limited follow-up time may be considered a limitation of this study. More time is needed to accurately report on the long-term course of the disease. However, after explantation the patients’ recovery has been swift, follow-up bloodwork indifferent and she has so far been doing well. Moreover, the primary aim of this study is to report a possible association between breast implants and CLL/SLL. This automatically presents the second limitation which is that the CLL/SLL may have been coincidental finding, combined with infiltration into the capsule which also is peculiar phenomenon. Unfortunately, we are not capable of fully understanding which mechanism initiated the disease in this patient. We do believe there is a strong association with the implants due to several reasons. Firstly, the patient first presented with symptoms surrounding her breast and did not experience any other symptoms located elsewhere. Secondly, both breasts contained siliconomas due to bilateral implant leakage possibly causing a chronic inflammatory response derailing the immune system. Thirdly, the lymphoma went into remission after explantation of both implants and so far no new symptoms have presented.

## 4 Conclusion

Primary lymphomas of the breast are extremely rare and most originate from the B-cell lineage. However, implant-associated breast lymphomas are increasingly being report and the majority are of T-cell lineage. Similar to most breast implant-associated lymphomas, explantation of the implant induced remission of the lymphoma. Long-term follow-up is advised consisting of CBC and immunophenotyping. MRI plays an important role in early stage imaging and diagnosis. In this report we present the case of a 62-year-old woman with bilateral CLL/SLL found in periprosthetic capsules.

## Declaration of Competing Interest

The authors declared no potential conflicts of interest with respect to the research, authorship, and publication of this article.

## Funding

The authors received no financial support for the research, authorship, and publication of this article.

## Ethical approval

This study was exempt from ethical approval by the institution’s medical ethics committee.

Code: 200316.

## Consent

Written informed consent was obtained from the patient for publication of this case report and accompanying images. A copy of the written consent is available for review by the Editor-in-Chief of this journal on request.

## Author contribution

Patrick Bletsis: study concept or design, data collection, data analysis or interpretation, writing the paper.

Laura Janssen: study concept or design, data collection, data analysis or interpretation, writing the paper.

Otto Visser: study concept or design, data analysis or interpretation, writing the paper.

Saskia Offerman: study concept or design, data collection, data analysis or interpretation, writing the paper.

Michiel Tellier: study concept or design, data collection, data analysis or interpretation.

Laurens Laterveer: study concept or design, data analysis or interpretation, writing the paper.

Peter Houpt: study concept or design, data collection, data analysis or interpretation, writing the paper.

## Registration of research studies

1 Name of the registry: Not required.
2 Unique identifying number or registration ID: Not required.
3 Hyperlink to your specific registration (must be publicly accessible and will be checked): Not required.

## Guarantor

Patrick P. Bletsis.

## Provenance and peer review

Not commissioned, externally peer-reviewed.

## References

[1] Hommes J., Mureau M.A.M., Harmsen M., Rakhorst H. (2015). ‘Which breast implant do I have?’; The importance of the Dutch Breast Implant Registry. Ned. Tijdschr. Geneeskd..

[2] de Jong D., Vasmel W.L.E., de Boer J.P., Verhave G., Barbé E., Casparie M.K. (2008). Anaplastic large-cell lymphoma in women with breast implants. JAMA.

[3] Swerdlow S.H., Campo E., Harris N.L., Jaffe E.S., Pileri S.A., Stein H.T.J. (2017).

[4] Zhu D., Fang C., Chen H., Wu C. (2015). Synchronous breast carcinoma and chronic lymphocytic leukemia in a Chinese young female: a rare combination. Int. J. Clin. Exp. Pathol..

[5] Agha R.A., Borrelli M.R., Farwana R., Koshy K., Fowler A.J., Orgill D.P. (2018). The SCARE 2018 statement: updating consensus surgical CAse REport (SCARE) guidelines. Int. J. Surg..

[6] Surov A., Holzhausen H.-J.-J., Wienke A., Schmidt J., Thomssen C., Arnold D. (2012). Primary and secondary breast lymphoma: prevalence, clinical signs and radiological features. Br. J. Radiol..

[7] Li S., Lee A.K. (2009). Silicone implant and primary breast ALK1-negative anaplastic large cell lymphoma, fact or fiction?. Int. J. Clin. Exp. Pathol..

[8] Cook P.D., Osborne B.M., Connor R.L., Strauss J.F. (1995). Follicular lymphoma adjacent to foreign body granulomatous inflammation and fibrosis surrounding silicone breast prosthesis. Am. J. Surg. Pathol..

[9] Said J.W., Tasaka T., Takeuchi S., Asou H., de Vos S., Cesarman E. (1996). Primary effusion lymphoma in women: report of two cases of Kaposi’s sarcoma herpes virus-associated effusion-based lymphoma in human immunodeficiency virus-negative women. Blood.

[10] Kraemer Dm, Tony H.-P.-P., Gattenlöhner S., Müller Jg. (2003). Lymphoplasmacytic lymphoma in a patient with leaking silicone implant. Haematologica.

[11] Nichter L.S., Mueller M.A., Burns R.G., Stallman J.M. (2012). First report of nodal marginal zone B-cell lymphoma associated with breast implants. Plast. Reconstr. Surg..

[12] Smith B.K., Gray S.S. (2014). Large B-cell lymphoma occurring in a breast implant capsule. Plast. Reconstr. Surg..

[13] Moling O., Piccin A., Tauber M., Marinello P., Canova M., Casini M. (2016). Intravascular large B-cell lymphoma associated with silicone breast implant, HLA-DRB1*11:01, and HLA-DQB1*03:01 manifesting as macrophage activation syndrome and with severe neurological symptoms: a case report. J. Med. Case Rep..

[14] Chen V.W., Hoang D., Clancy S. (2018). Breast implant-associated bilateral B-cell lymphoma. Aesthetic Surg. J..

[15] Lee Y.-S., Filie A., Arthur D., Fojo At, Jaffe Es. (2015). Breast implant-associated anaplastic large cell lymphoma in a patient with Li-Fraumeni syndrome. Histopathology.

[16] Topalovski M., Crisan D., Mattson J.C. (1999). Lymphoma of the breast. A clinicopathologic study of primary and secondary cases. Arch. Pathol. Lab. Med..

[17] Tsimberidou A.-M.-M., Wen S., McLaughlin P., O’Brien S., Wierda W.G., Lerner S. (2009). Other malignancies in chronic lymphocytic leukemia/small lymphocytic lymphoma. J. Clin. Oncol..

[18] Ng D., Toure O., Wei M.-H.-H., Arthur D.C., Abbasi F., Fontaine L. (2007). Identification of a novel chromosome region, 13q21.33-q22.2, for susceptibility genes in familial chronic lymphocytic leukemia. Blood.

[19] Yamamoto J.F., Goodman M.T. (2008). Patterns of leukemia incidence in the United States by subtype and demographic characteristics, 1997-2002. Cancer Causes Control.

[20] Brown J.R., Neuberg D., Phillips K., Reynolds H., Silverstein J., Clark J.C. (2008). Prevalence of familial malignancy in a prospectively screened cohort of patients with lymphoproliferative disorders. Br. J. Haematol..

[21] Goldin L.R., Lanasa M.C., Slager S.L., Cerhan J.R., Vachon C.M., Strom S.S. (2010). Common occurrence of monoclonal B-cell lymphocytosis among members of high-risk CLL families. Br. J. Haematol..

[22] Wiernik P.H., Ashwin M., Hu X.P., Paietta E., Brown K. (2001). Anticipation in familial chronic lymphocytic leukaemia. Br. J. Haematol..

[23] Jones S.J., Voong J., Thomas R., English A., Schuetz J., Slack G.W. (2017). Nonrandom occurrence of lymphoid cancer types in 140 families. Leuk. Lymphoma.

[24] Diver W.R., Teras L.R., Gaudet M.M., Gapstur S.M. (2014). Exposure to environmental tobacco smoke and risk of non-Hodgkin lymphoma in nonsmoking men and women. Am. J. Epidemiol..

[25] Talibov M., Auvinen A., Weiderpass E., Hansen J., Martinsen J.-I.-I., Kjaerheim K. (2017). Occupational solvent exposure and adult chronic lymphocytic leukemia: No risk in a population-based case-control study in four Nordic countries. Int. J. Cancer.

